# Effect of slow-release fertilizer on soil fertility and growth and quality of wintering Chinese chives (*Allium tuberm* Rottler ex Spreng.) in greenhouses

**DOI:** 10.1038/s41598-021-87593-1

**Published:** 2021-04-13

**Authors:** Cheng Wang, Jian Lv, Jianming Xie, Jihua Yu, Jing Li, Jing Zhang, Chaonan Tang, Tianhang Niu, Bakpa Emily Patience

**Affiliations:** College of Horticulture, Gansu Agricultural University, Yingmen Village, Anning District, Lanzhou, 730070 China

**Keywords:** Plant sciences, Agroecology, Agroecology

## Abstract

To avoid the negative impact of excessive fertilization on vegetable production, a decreased fertilization experiment was conducted in a multi-layer covered plastic greenhouse in 2017 to 2018. Treatments included no fertilizer (CK), traditional fertilization (TF), slow-release fertilizers (SRF), and decreased fertilization with slow-release fertilizers (DSRF). Results showed that the SRF and DSRF increased leaf length (13% and 8.3%) and chlorophyll content (7.1% and 8.2%) of Chinese chives compared to TF. Similarly, DSRF was found to increase the accumulation of dry matter accumulation of roots (22%) and the dry matter accumulation of shoots (36%) of Chinese chives. Flavonoid, soluble sugar, and soluble protein content were enhanced by 18%, 8.5%, and 4.6%, respectively, in DSRF compared to TF. Nitrate content of the SRF and SRFR decreased significantly by 26% and 35%, respectively. In addition, there was a significant increase in soil nutrient and enzyme activity in the middle and late harvest of Chinese chives under DSRF compared to TF, and there was a high correlation between soil nutrients and the quality of Chinese chives. The available P and total P content significantly differed among the different greenhouse soil samples, and this significantly affected the quality of Chinese chives. The content of available P and total P in greenhouse soil was 125.07 g kg^−1^ and 1.26 mg kg^−1^, respectively. Optimal quality was obtained. Hence, the application of DSRF promoted the growth of Chinese chives and improved soil fertility, thereby enhancing the productivity and quality of Chinese chives.

## Introduction

Chinese chives (*Allium tuberosum* Rottler ex Spreng.) are a hardy perennial plant widely cultivated in China^1^. The optimal temperature for the growth of the above ground part of Chinese chives is 13–20 ℃, and the tolerable low temperature is – 4 to 5 ℃. When the temperature drops to − 6 to 7 ℃, the leaves begin to wither gradually, and nutrients gradually return to the roots to ensure normal growth through the winter^2^. There is a long history of Chinese chives cultivation for culinary and medicinal use^3^. It has a unique mild garlic flavor and is rich in vitamins, fiber, and sulfur compounds with antibiotic properties^4^. Chinese chives not only have high nutritional value, but also have antioxidant, antibacterial, anti-inflammatory, and anti-cancer effects as a traditional medicinal material^5–7^. Therefore, they have become increasingly economically valuable^8^.

As a facility cultivation mode with a simple structure, a convenient operation, a low investment requirement, energy saving, and environmental protection, a multi-layer covered plastic greenhouse is of great significance for the overwintering production of cold-tolerant vegetables and early spring vegetable cultivation. Wushan, China, is known as the "Hometown of Chinese Chives". Due to its unique sandy soil and unique overwintering planting methods in multi-layer covered plastic greenhouse, the quality of Chinese chives in Wushan is superior. Therefore, exploring the factors that affect the growth and quality of Wushan Chinese chives is of great significance to improve their yield and quality. However, excessive fertilization was more common in the production of wintering Chinese chives in Wushan, which severely restricts the increase in the output of Wushan Chinese chives and the progress in their high-quality production. Excessive use of chemical fertilizers, unbalanced fertilization, and improper fertilization methods lead to low nitrogen (N) efficiency in the soil, as well as leaching, volatilization, and nitrification of N, causing a series of environmental hazards and economic losses^9–13^. Similarly, the substantial increase in N fertilizer input will not bring about a corresponding increase in crop yields, and will increase unnecessary labor^14,15^. Therefore, optimizing fertilization management to improve soil fertility is essential to meet plant nutrient requirements, increase yield and quality, improve rural economy and maintain sustainable agricultural development, and achieve environmentally friendly agriculture^16^. In order to better meet the nutrient requirements of plants, slow-release fertilizers (SRF) have been developed to slow down or even control the release rate of nutrients into the soil^17,18^. The use of SRF shows an advantage over ammonium nitrate and urea, and its relative performance varies with rainfall, fertilization level, and soil quality^19^. Compared with traditional fertilizers (TFs), slow-release fertilizers can maintain or even increase yields even with the same or reduced dosage^17^. The synchronicity of the time and rate of the nutrient release of SRF can meet the nutrient requirements of plants and minimize the loss of fertilizer nutrients^20^, and one application can meet the nutrient requirements of the entire growth period of the crop^21^. Although, compared with TF, the application of SRF in agricultural production is limited due to higher production costs, but the application of SRF reduces the input cost and the frequency of fertilization, and is a viable alternative method to avoid the excessive use of fertilizers^22^. In addition, soil nutrients are a key factor affecting crop quality^23^. The amount of fertilizer supplied to plants should be effective enough to increase the effective nutrients of the soil absorbed by the plants to increase yield and quality^24^. Studies have shown that the nutrients in the soil, especially phosphorus (P) and potassium (K), have an important impact on yield and quality^25,26^. According to the index of abundance and deficiency of available K content in vegetable soil, the appropriate content of available K in soil is 150–250 mg·kg^−1^, more than 350 mg·kg^−1^ is excessive^27^. The content of available P in vegetable field soil exceeds the environmental threshold (P 80 mg kg^−1^), and obvious leaching of P occurs in the profile^28^. Huang et al. found that reducing the application of P fertilizers appropriately would increase the yield and quality of tomatoes^29^. Giroto et al.^30^ showed that application of SRF can increase soil effective nutrient content and improve soil fertility. Therefore, it may be possible to improve the quality of crops by applying SRF to improve soil nutrients. There have been a lot of studies on soil, fertilization and fruit quality of fruit trees^31,32^. There are also some reports on the correlation between soil nutrients and fruit quality in local apple orchards, multivariate statistical analysis and optimization schemes, etc^33,34^. Wang et al. studied the correlation between Xinjiang orchard soil and fruit quality factors and determined the range of soil nutrients for the best fruit quality through response surface analysis^23^. However, there have been few reports about the relationship between soil nutrients and quality in greenhouse vegetables. Most research focuses on the unilateral application of fertilizers to improve soil fertility and quality, and has not systematically and comprehensively studied the relationship between fertilizer-soil nutrients-quality.

China's reports on SRF mainly focus on the yield and nutrient utilization of field crops such as rice, corn, and wheat^35–37^, while foreign research on SRF mainly focuses on seedling production^38^. The above-ground growth of Chinese chives in greenhouses in winter mainly depends on the above-ground nutrients returning to the roots and the nutrients stored in the roots during the rooting period, but the mechanisms underlying this wintering growth remain largely unknown. There is no research data on the effects of SRF and decreased fertilization on the growth, quality, and soil fertility of wintering Chinese chives in greenhouse, and there are few reports on the effects of soil nutrients on vegetable quality. We hypothesized that the application of SRF and decreased fertilization would (i) promote the growth of Chinese chives and improve the quality of Chinese chives and (ii) increase effective soil nutrients and related enzyme activities, thereby (iii) improving soil fertility and increasing the productivity of wintering Chinese chives. The purpose of this study was to study the effects of SRF and decreased fertilization on the growth, quality, soil nutrients, and related enzyme activities of wintering Chinese chives in multi-layer (four-layer) covered plastic greenhouses, and to explore the dormant growth mechanism of Chinese chives overwintering. In addition, we analyzed the multiple correlation between quality and greenhouse soil nutrients, and used a variety of statistical analysis methods to explore an appropriate soil nutrient scheme for planting high-quality Chinese chives.

## Results

### Morphological parameters

**Figure 1:**
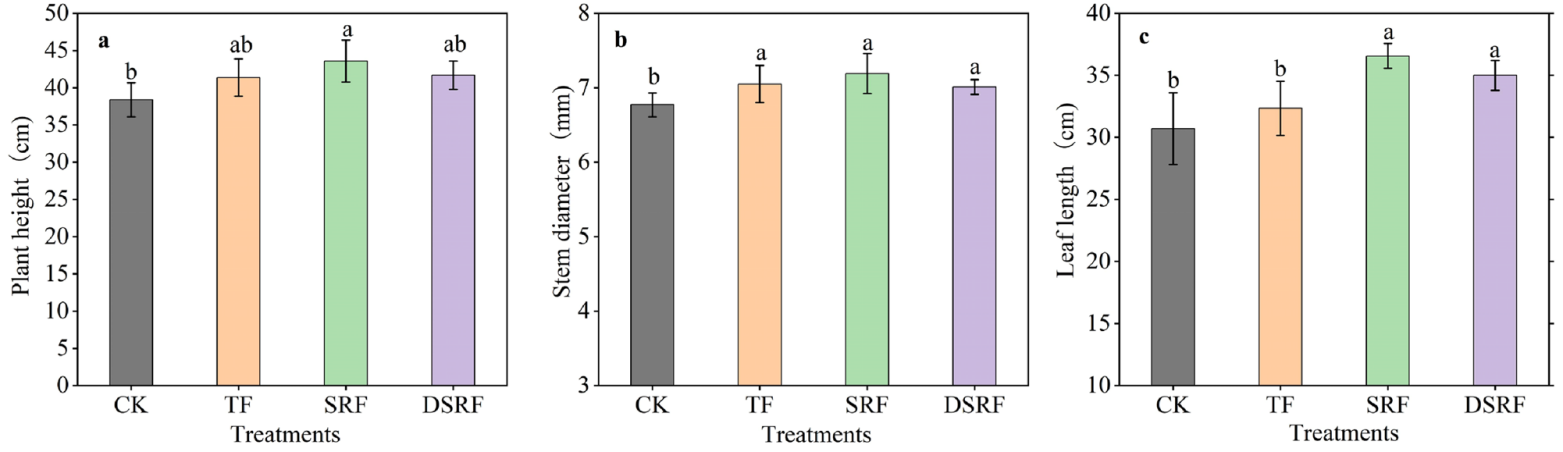
Plant height (**a**), stem diameter (**b**), and leaf length (**c**) of wintering Chinese chives as affected by different fertilizer treatments. Lowercase letters indicate significant differences among different fertilization treatments (*p* < 0.05). *CK* no fertilizer, *TF* traditional fertilization, *SRF* traditional fertilization with slow-release fertilizer, *DSRF* decreased fertilization with slow-release fertilizer.

The SRF and DSRF treatments significantly improved the leaf length of wintering Chinese chives cultivated greenhouses during winter by 13% and 8.3%, respectively, compared to TF (Fig. 1c), but had no significant effect on the plant height and stem diameter of Chinese chives (Fig. 1a,b).

**Figure 2:**
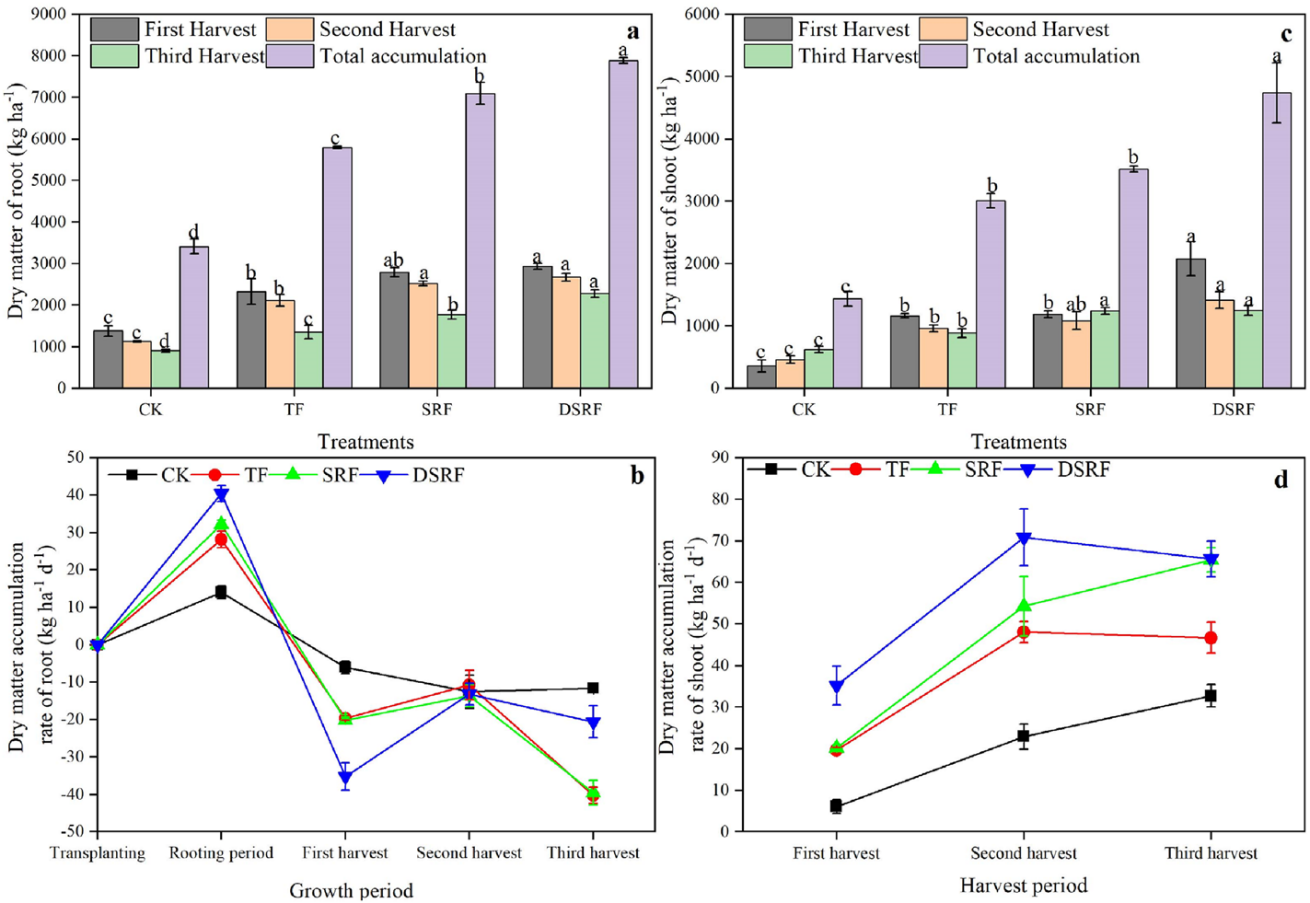
Dry matter accumulation of the roots (**a**), dry matter accumulation rate of the roots (**b**), dry matter accumulation of shoots (**c**), and dry matter accumulation rate of the shoots (**d**) of wintering Chinese chives as affected by different fertilizer treatments. Lowercase letters indicate significant differences among different fertilization treatments (*p* < 0.05). *CK* no fertilizer, *TF* traditional fertilization, *SRF* traditional fertilization with slow-release fertilizer, *DSRF* decreased fertilization with slow-release fertilizer.

The application of SRF can significantly promote the accumulation of dry matter in the roots of Chinese chives, and the dry matter quality of the roots in each harvest period gradually decreases (Fig. 2a). The total accumulation of root dry matter of SRF and DSRF were significantly increased by 22% and 36%, respectively, as compared to TF. The shoot dry matter accumulation of DSRF was significantly higher than that of TF in each harvest period, and the total accumulation of shoots was significantly increased by 58% (Fig. 2c). In terms of the dry matter accumulation rate, during the rooting period, the dry matter accumulation rate of the roots of the SRF and DSRF showed an upward trend, which was significantly higher than that of TF (Fig. 2b). After entering the harvest period, the accumulation rate of dry matter in the roots of SRF and DSRF showed a negative growth trend (Fig. 2b), while the accumulation rate of dry matter in the shoots increased rapidly significantly higher than that of TF (Fig. 2d).

### Photosynthetic pigment

**Figure 3:**
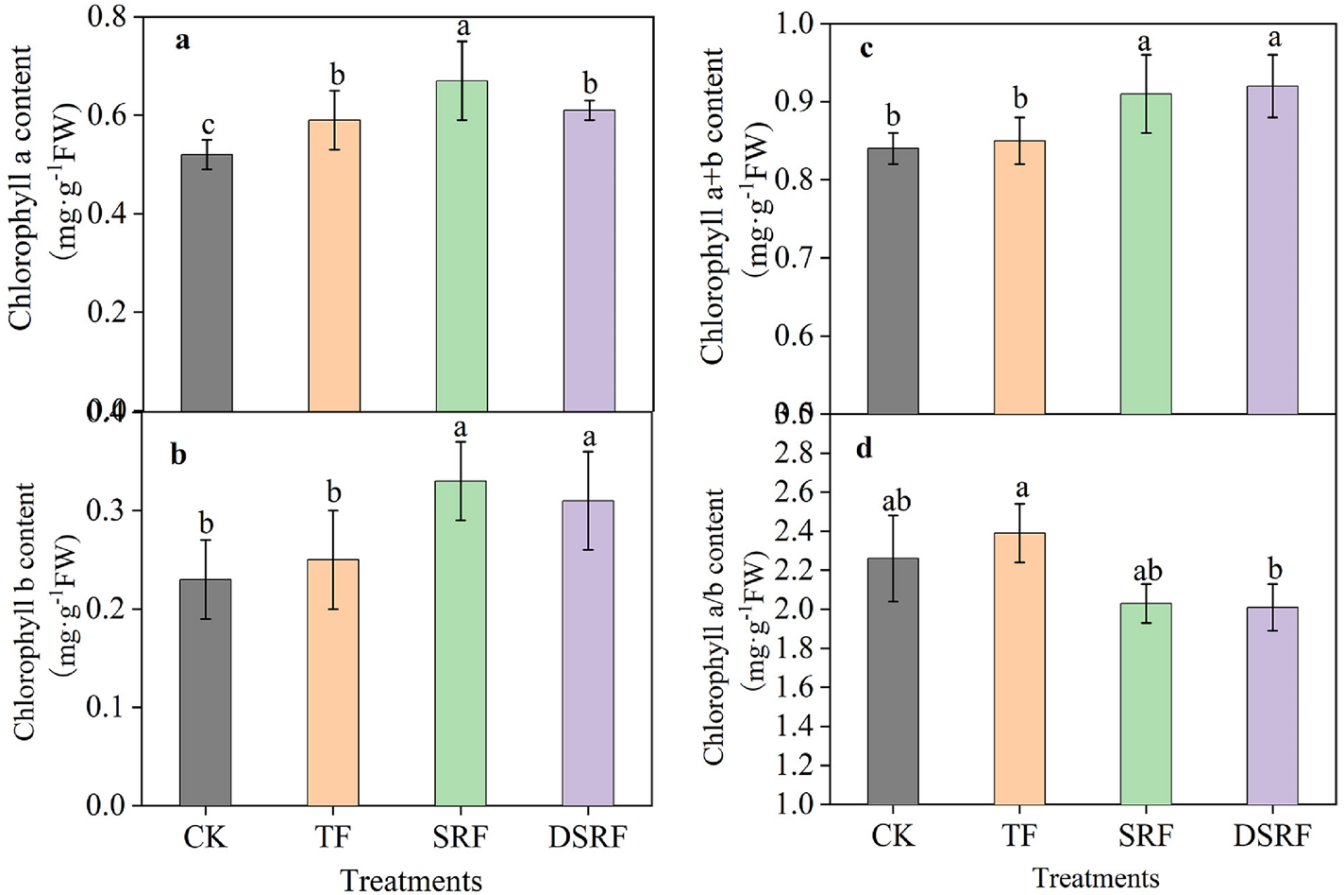
Chlorophyll a (**a**), chlorophyll b (**b**), chlorophyll a + b (**c**), and chlorophyll a/b (**d**) of wintering Chinese chives as affected by different fertilizer treatments. Lowercase letters indicate significant differences among different fertilization treatments (*p* < 0.05). *CK* no fertilizer, *TF* traditional fertilization, *SRF* traditional fertilization with slow-release fertilizer, *DSRF* decreased fertilization with slow-release fertilizer.

The application of SRF was found to significantly promote the increase of chlorophyll content of Chinese chives leaves (Fig. 3). The chlorophyll a content of SRF was significantly increased by 14%, while DSRF had no significant effect on it (Fig. 3a), as compared to TF. The chlorophyll b and chlorophyll a + b content of the SRF and DSFR were significantly increased by 32% and 24%, and by 7.1% and 8.2%, respectively, as compared to TF (Fig. 3b,c). However, the chlorophyll a/b content in DSRF was significantly lower than that in TF (Fig. 3d).

### Nutritional quality

**Figure 4:**
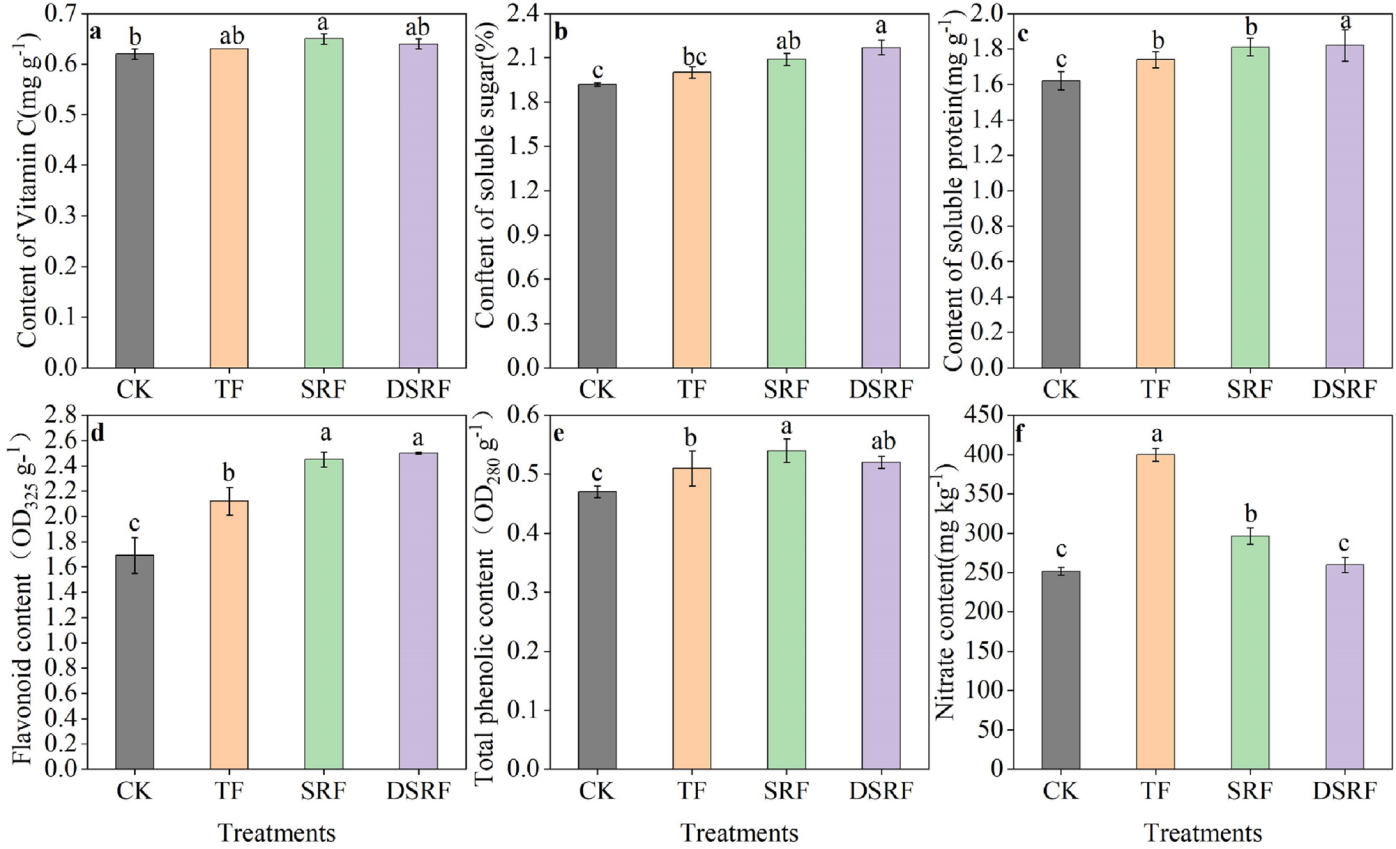
Vitamin C (**a**), soluble sugar (**b**), soluble protein (**c**), flavonoids (**d**), total phenols (**e**), and nitrate (**f**) of wintering Chinese chives as affected by different fertilizer treatments. Lowercase letters indicate significant differences among different fertilization treatments (*p* < 0.05).* CK* no fertilizer, *TF* traditional fertilization, *SRF* traditional fertilization with slow-release fertilizer, *DSRF* decreased fertilization with slow-release fertilizer.

The SRF and DSRF treatments significantly improved the flavonoid content of Chinese chives cultivated in greenhouses during winter by 16% and 19%, respectively, compared to TF (Fig. 4d). However, DSRF had no significant effects on vitamin C and total phenol content were not significant (Fig. 4a,e). Soluble sugar and soluble protein content in the SRF treatment were not significantly increased, whereas the SRFR treatment had a significantly higher soluble sugar (8.5%) and soluble protein (4.6%) content compared to TF (Fig. 4b,c). Nitrate content of the SRF and SRFR treatments decreased significantly by 26% and 35%, respectively, compared to TF (Fig. 4f).

### Soil nutrient

**Figure 5:**
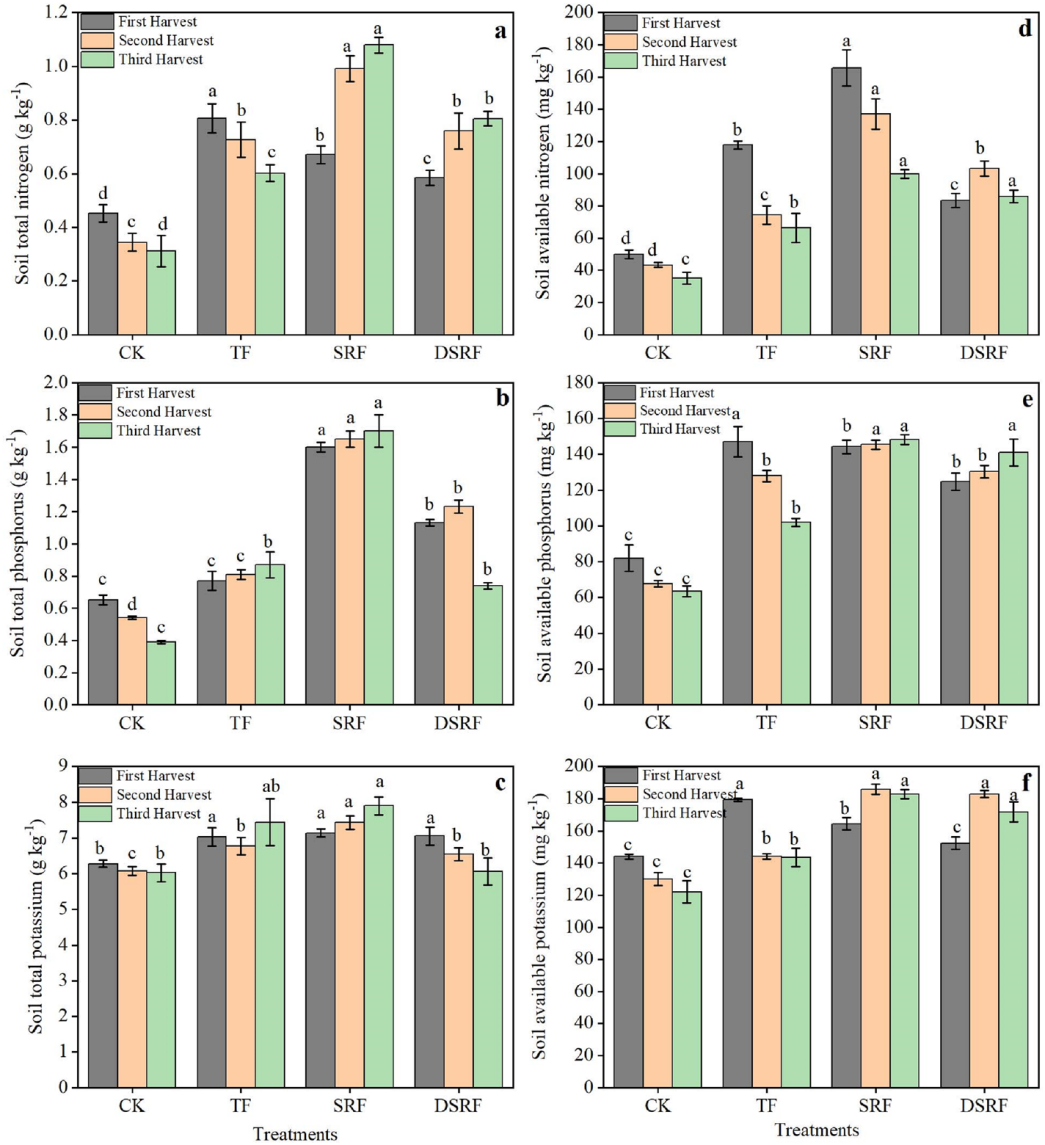
Soil total nitrogen (**a**), total phosphorus (**b**), total potassium (**c**), available nitrogen (**d**), available phosphorus (**e**), and available potassium (**f**) of wintering Chinese chives as affected by different fertilizer treatments. Lowercase letters indicate significant differences among different fertilization treatments (*p* < 0.05). *CK* no fertilizer, *TF* traditional fertilization, *SRF* traditional fertilization with slow-release fertilizer, *DSRF* decreased fertilization with slow-release fertilizer.

The application of SRF significantly improved soil fertility in greenhouses in winter (Fig. 5). The soil total N content of SRF and DSRF had significantly lower than TF in the early stage of Chinese chive harvest; however, the soil total N of the SRF and DSRF treatments was 58% and 34% greater, respectively, than that of the TF treatment in the later stage of the Chinese chive harvest (Fig. 5a). Compared to the TF treatment, the soil total P of DSRF was significantly increased by 50% at the early and mid-harvest stages of Chinese chives (Fig. 5b). The soil total K of the SRF treatment was significantly greater than those of TF in the middle and late harvest of Chinese chives, whereas the SRFR treatment had no significant effect (Fig. 5c). The soil available N of SRF in the harvest of Chinese chives was 63% greater than that of the TF, and that of DSRF increased 35% in the middle and late harvest (Fig. 5d). The soil available P and available K of SRF and DSRF in the early stage of harvest were lower than those of the TF, but the soil available P and available K in the late stage of harvest were increased by 29% and 38% for SRF and by 28% and 34% for DSRF, respectively (Fig. 5e,f).

### Soil enzyme activity

**Figure 6:**
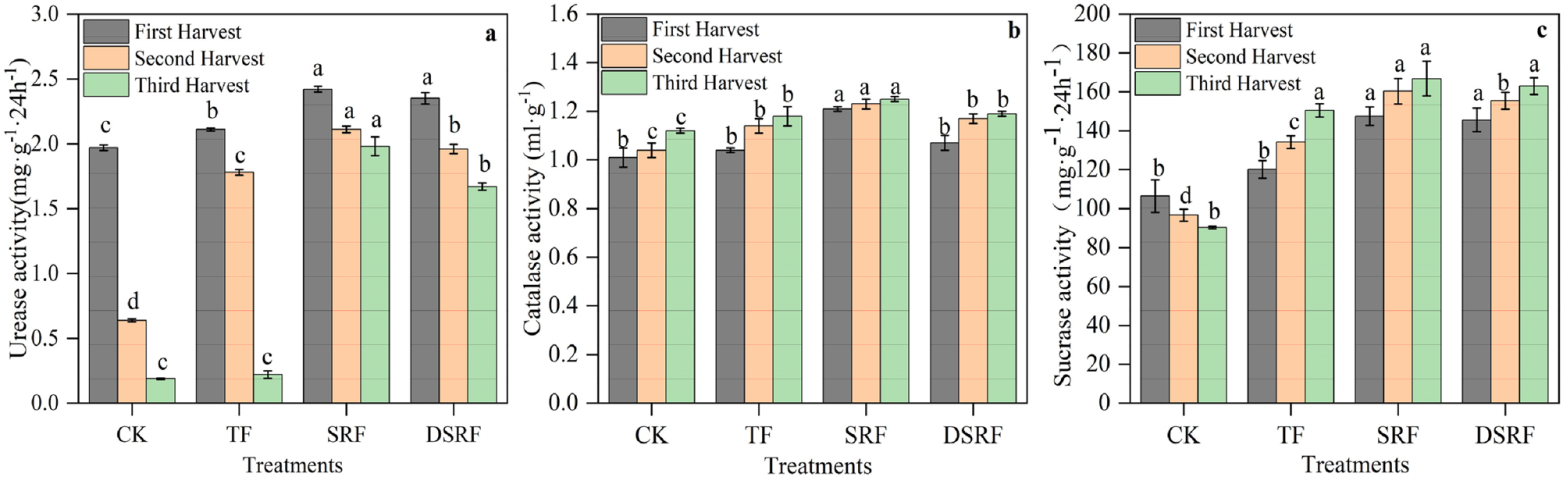
Soil urease activity (**a**), soil catalase activity (**b**), and soil sucrase activity (**c**) of wintering Chinese chives as affected by different fertilizer treatments. Lowercase letters indicate significant differences among different fertilization treatments (*p* < 0.05). *CK* no fertilizer, *TF* traditional fertilization, *SRF* traditional fertilization with slow-release fertilizer, *DSRF* decreased fertilization with slow-release fertilizer.

The enzyme activity found 0–20 cm deep in the soil was significantly affected by fertilization. The soil urease and sucrase activities during the harvest of Chinese chives was increased by 40% and 17% for SRF, respectively, and by 24% and 5.2% for DSRF, respectively (Fig. 6a,c). The soil catalase activity of SRF treatment was 11% greater than that the TF treatment during the harvest of Chinese chives, while DSRF had no significant effect on it during the entire harvest of Chinese chives (Fig. 6b).

### Correlation analysis of soil enzyme activity and soil nutrients

**Figure 7:**
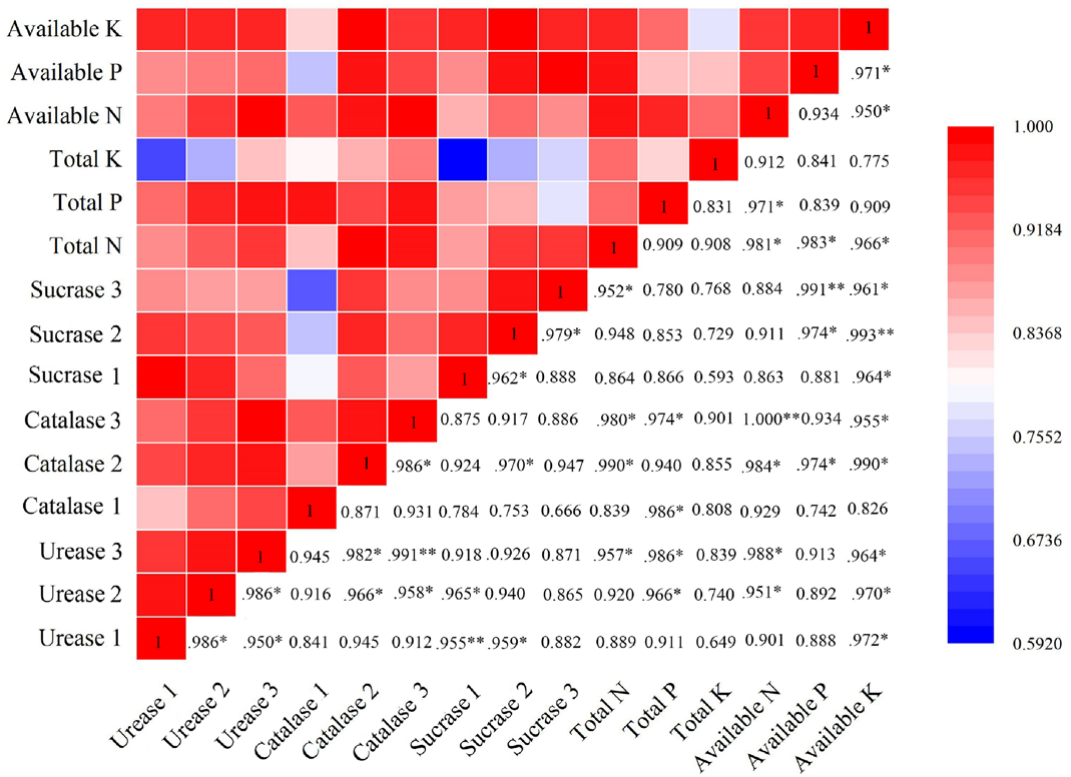
Heat map of Pearson's correlation coefficient matrix between soil enzyme activity and soil nutrients. Urease 1, Catalase 1, Sucrase 1 represent soil enzymes during the first harvest. Urease 2, Catalase 2, Sucrase 2 represent soil enzymes during the second harvest. Urease 3, Catalase 3, Sucrase 3 represent soil enzymes during the third harvest. The values in the figure are Pearson's correlation coefficient. * and ** denote correlation coefficients that are significant at *p* < 0.05 and 0.01 level, respectively. Heat map of Pearson's correlation coefficient matrix was produced by OriginPro 2019b 32Bit.

The correlation between soil nutrients and soil enzymes in a multi-layer covered plastic greenhouse was analyzed ((Fig. 7). The results showed that, in the early stage of Chinese chive harvest, soil urease, soil catalase, and soil sucrase were significantly correlated with the content of available K, total P, and available K, respectively (*p* < 0.05). In the middle of Chinese chive harvest, both soil urease and soil catalase showed a significant correlation with soil available N and available K (*p* < 0.05). There was a significant correlation between soil sucrase and available P (*p* < 0.05), and a significant correlation between soil sucrase and available K at *p* < 0.01. In the end of the Chinese chive harvest, soil total N, total P, available N, and available K had great effects on soil urease and catalase activities. Available N and available P showed a significant correlation with catalase and sucrase, respectively (*p* < 0.01), and total N and available K had a significant correlation with catalase and sucrase at *p* < 0.05.

### Analysis on the difference of soil nutrients under different fertilization treatments

**Figure 8:**
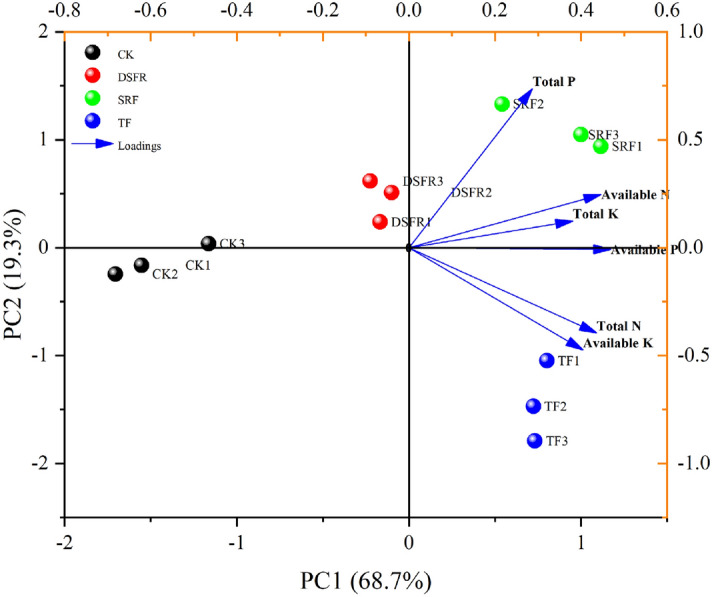
Principal component analysis (PCA) of different fertilization treatments and soil nutrients, including PCA score scatter plot and PCA loading plot.

To comprehensively reflect the effects of different fertilization treatments on greenhouse soil nutrients, we conducted principal component analysis (PCA) on the measured six soil nutrient factors (Fig. 8). It could be obtained from the two-dimensional PCA in Fig. 8, the 4 different fertilization treatments and 6 kinds of soil nutrients formed corresponding groupings. Principle component analysis (PCA) is meant to address multicollinearity between multiple covariates. To determine the number of principle components (PCs) to retain, one should use the eigen values. In principle, the PCs that should be retained are those with eigenvalues > 1. The results showed that the first and second principal components captured most of the variation within the different fertilization treatments. The sum of the first two principal components reached 88.0%, of which PC1 represented 68.7% of the total variance, and PC2 represented 19.3% of the total variance. The first principal component can be used to distinguish between traditional fertilizer rate and reduced fertilization, and the second principal component can be used to distinguish between ordinary chemical fertilizers and slow-release fertilizers. In addition, it can be seen from the loading map that available P and total P have strong loading with the first principal component and the second principal component, respectively, which can be used as representative factors to reflect the differences of soil nutrients in different fertilization treatments.

### Analysis on correlation between representative soil nutrients and wintering Chinese chives quality

**Figure 9:**
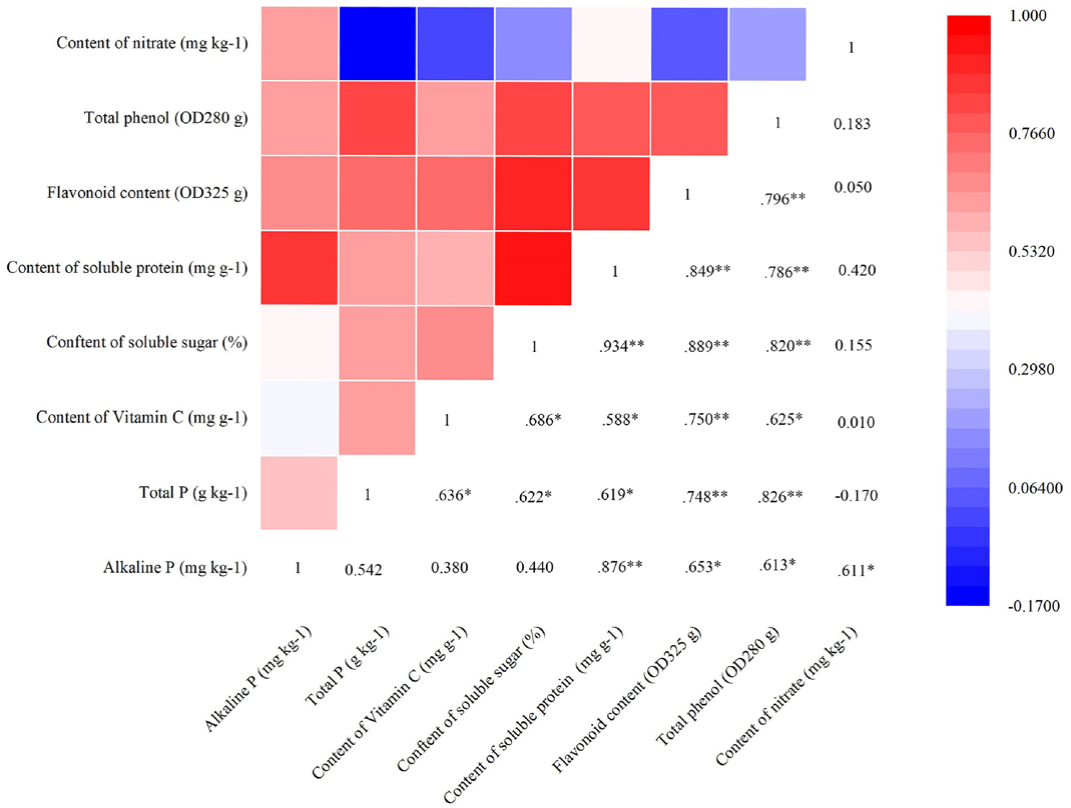
Heat map of Pearson's correlation coefficient matrix between the wintering Chinese chives quality variables and available P and total P. The values in the figure are Pearson's correlation coefficient. * and ** denote correlation coefficients that are significant at *p* < 0.05 and 0.01 level, respectively. Heat map of Pearson's correlation coefficient matrix was produced by OriginPro 2019b 32Bit.

The correlation analysis between representative soil nutrients and Chinese chive quality showed that the content of soil available P had a significant correlation with the soluble protein content of Chinese chives at *p* < 0.01, and a significant correlation with the content of flavonoids, total phenols, and nitrate at *p* < 0.05 (Fig. 9). The content of soil total P had a significant correlation with flavonoids, and total phenol content of Chinese chives (*p* < 0.01), and it had a significant correlation with vitamin C, soluble sugar, and soluble protein (*p* < 0.05). Furthermore, available P and total P had no significantly correlated with vitamin C, soluble sugar, and nitrate content was observed, respectively.

### Optimizing soil nutrients to improve the quality of wintering Chinese chives

Based on the above response of Chinese chives quality to available P and total P, it was indicated that different soil nutrients have different effects on the quality of Chinese chives. Therefore, a variety of statistical analysis methods were used to further explore the relationship between soil nutrients and the quality of Chinese chives to clarify the range of nutrients for high-quality Chinese chives in greenhouse soil. To optimize the content of soluble protein (Y1), flavonoids (Y2), and total phenols (Y3) of Chinese chives, the optimal range of soil available P (X1) and total P (X2) content was analyzed by response surface method. The results showed that the main reason for the fitting model to explain the variation of Chinese chive quality was the linear, quadratic and cross product effects of available P and total P contents. The fitted response surface models were as follows:

$${\text{Y1 = 1}}{.81} + {9}{\text{.27X}}_{{1}} + {0}{\text{.01 X}}_{{2}} + {0}{\text{.05X}}_{{1}} {\text{X}}_{{2}} - {0}{\text{.07X}}_{{1}}^{{2}} - {0}{\text{.03X}}_{{2}}^{{2}}$$

(Fig. 10a) and

$${\text{Y2 }} = { 2}{\text{.47}} + {5}{\text{.03X}}_{{1}} + {0}{\text{.01X}}_{{2}} + {0}{\text{.28X}}_{{1}} {\text{X}}_{{2}} - {0}{\text{.30X}}_{{1}}^{{2}} - {0}{\text{.14X}}_{{2}}^{{2}}$$

**Figure 10:**
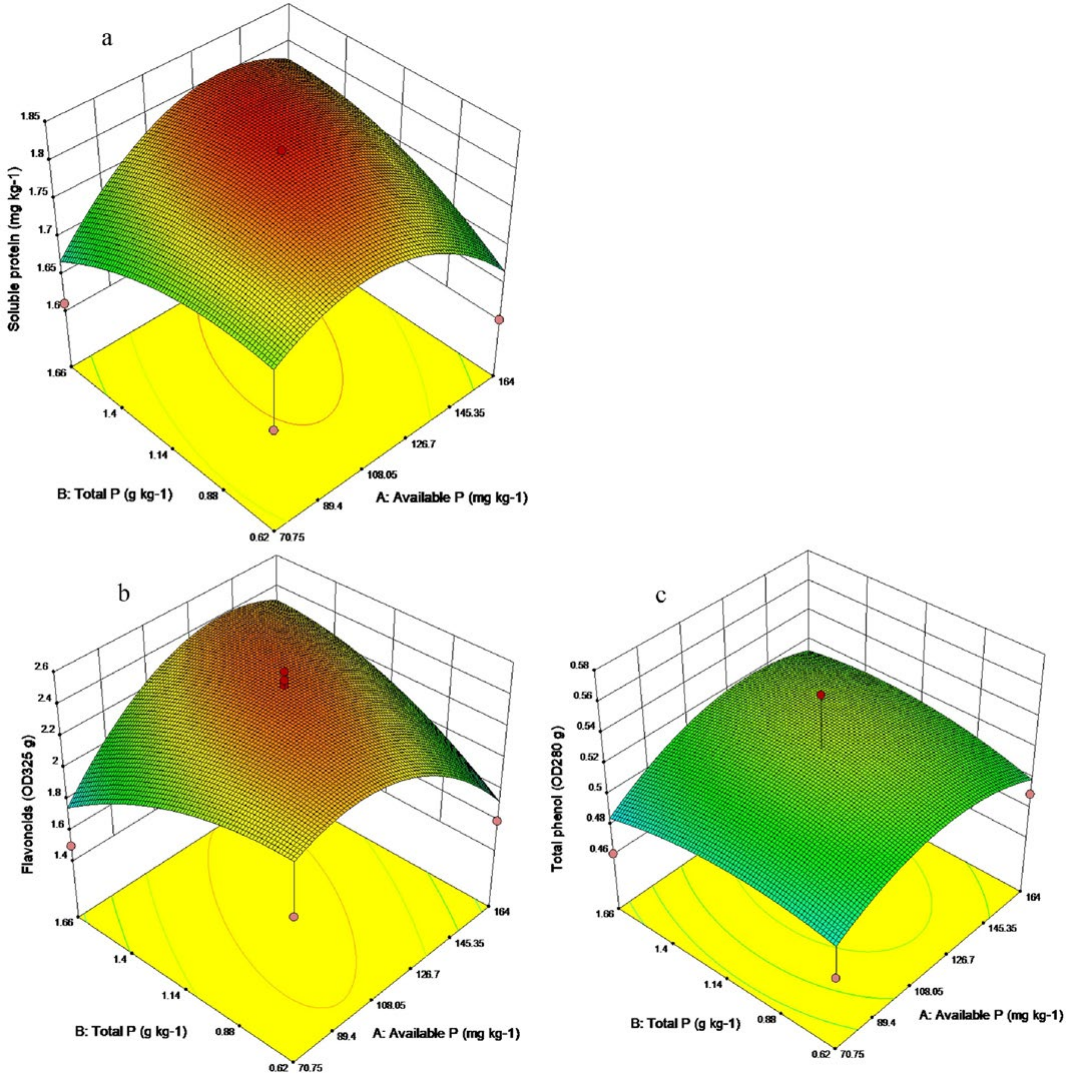
Response surface analysis of Chinese chives quality to soil nutrients. (**a**) Effect of interaction between the available P (X1) and total P (X2) content on the soluble sugar content (Y1). (**b**) Effect of interaction between the available P (X1) and total P (X2) content on the flavonoid content (Y2). (**c**) Effect of interaction between the available P (X1) and total P (X2) content on the total phenol content (Y3).

(Fig. 10b) and

$${\text{Y3 }} = { 0}{\text{.53}} + {9}{\text{.27X}}_{{1}} + {1}{\text{.77X}}_{{2}} + {5}{\text{.00X}}_{{1}} {\text{X}}_{{2}} - {0}{\text{.02X}}_{{1}}^{{2}} - {9}{\text{.25X}}_{{2}}^{{2}}$$

(Fig. 10c). The prediction results of the model showed that the best quality of Chinese chive (1.84 mg kg^−1^ soluble protein, 2.46 OD_325_ g flavonoids, and 0.53 OD_280_ g total phenol) could be obtained when the contents of available P and total P in greenhouse soil were 125.07 mg kg^−1^ and 1.26 g kg^−1^, respectively. The results show that a reasonable application of P fertilizer can improve the quality of wintering Chinese chives in greenhouse.

## Discussion

Dry matter is the accumulation of photosynthetic substances and absorbed nutrients in plants, which affect the formation of crop yields. Carreres et al.^39^ found that SRF could promote the root development and nutrient absorption capacity of crops. The growth of Chinese chives in greenhouses in the winter production period mainly depends on the nutrients stored in the roots before winter. In the present study, the SRF and DSRF increased leaf length (13% and 8.3%) and chlorophyll content (7.1% and 8.2%) of Chinese chives compared to TF (Figs. 1c, 3c). DSRF was found to increase the accumulation of dry matter accumulation of roots (22%) and the dry matter accumulation of shoots (36%) of Chinese chives (Fig. 2a,c). Frequent irrigation in vegetable production leads to a large amount of N leaching loss, denitrification, and volatilization in ordinary fertilizers^40^, resulting in a low N recovery rate in the soil–plant system^41^ and insufficient N supply in the later stage of plant growth, which in turn leads to plant dry matter synthesis ability declines. However, SRF can slow down the decomposition of N into ammonia, reduce the release rate of fertilizer N, synchronize N supply with crop N demand, and maintain a sustained and stable nutrient supply during the crop growing season, thereby improving the ability of plant dry matter synthesis and increasing its accumulation^42–45^. Simultaneously, we discussed the accumulation rate of dry matter of Chinese chives in each growth period to clarify the dormant growth mechanism of Chinese chives overwintering (Fig. 2b,d). When the monthly average temperature drops below 2 °C, the nutrients in the leaves and sheaths of Chinese chives begin to flow back, and are stored in the base of the sheath, rhizomes and roots. The leaves begin to wither and the plants enter dormancy, which is called " Back to the root" ^46^. That is, during the root raising period, we found that the dry matter accumulation rate of the roots of SRF and DSRF showed an upward trend. After entering the harvest period, the roots provide the main nutrient supply. The accumulation rate of dry matter in the roots of SRF and DSRF showed a negative growth trend, and the accumulation rate of dry matter in the shoots increased rapidly, which promoted the transportation of nutrients from the roots to the shoot, accelerated the accumulation of dry matter in the shoots, and improved the wintering productivity of the Chinese chives.

Excessive and insufficient fertilization will have adverse effects on the environment and the growth and quality of crops^47^. Research has shown that K improves the metabolism of carbohydrates and nitrogen, thereby improving the quality of crops^48–50^. In the present study, the DSRF treatment significantly increased the content of soluble sugar (8.5%), soluble protein (4.6%), and flavonoids (18%) in leaves (Fig. 4). This may be due to the fact that the DSRF treatment reduced nutrients by 31% under the condition of balanced fertilization and appropriately increased the input of K. The reasonable combination and slow release of N, P, and K nutrients met the physiological requirements of Chinese chives in different growth periods, thereby improving their nutritional quality. In addition, SRF could significantly reduce the accumulation of nitrate in their leaves. This result may be because SRF with urease inhibitors and nitrification inhibitors can adjust the nitrification of ammonium nitrogen, thereby adjusting the ratio of ammonium to nitrate and reducing the volatilization and nitrification of ammonium nitrogen, which increases the utilization of nitrogen and thus reduces the nitric acid in Chinese chive leaves^51^.

Fertilization directly affects soil quality and its productivity, while soil quality is the result of the comprehensive influence of many factors, such as soil nutrients, physical and chemical properties, soil structure and texture, etc.^52^. Maintaining good physical, chemical, and biological properties of the soil is essential for ensuring soil safety, maintaining high crop yields, and promoting the rural economy^16^. Excessive use of chemical fertilizers can distort soil composition and properties such as fertility and integrity^22^. Our study showed SRF increased the effective nutrient content of the soil (Fig. 5). SRF increased the content of soil total N, total P, and available N during the harvest of Chinese chives and maintained a high supply capacity of soil available P and available K in the middle and later stages of harvest. DSRF maintained a high P supply capacity in the early stage of harvest and significantly increased the content of soil total N, available N, available P, and available K in the later stage of harvest. The production period of Chinese chives in greenhouses is longer in winter, during which there is no fertilization and no irrigation, which requires the continuous supply of sufficient nutrients stored in the roots and available nutrients in the soil during the production period. The results of this study showed that the application of SRF maintains a higher soil nutrient supply capacity than TF and prolongs the soil nutrient supply time. Ordinary fertilizers will be released in large amounts of nutrients in the early stage, and a large amount of nutrients cannot be absorbed and utilized by plants and accumulate in the soil. This may cause an excessively high soluble salt concentration around the root system, causing difficulty for plant roots to absorb nutrients and affecting plant growth and development. However, while SRF reduced the amount of fertilizer, the slow release of nutrients adapted to the nutrient needs of crops, and the nutrients were not released in large quantities after being applied to the soil. It slowly supplied nutrients according to different growth periods of crops, and improved the soil fertilizer supply capacity in the later growth stage of Chinese chives^53^.

Soil quality is closely related to soil physical and chemical properties and soil biological characteristics. Soil enzyme activity mainly reflects soil microbiological activity and soil nutrient transformation ability. It is closely related to soil types, crops, and fertilizers, the mode of production, and the amount of fertilization. Its activity directly affects the soil microbial environment and then the soil fertility^54^. Our results demonstrate that SRF increased the activities of soil-related enzymes during the harvest of Chinese chives, more so than the TF treatment (Fig. 6). DSRF significantly increased soil urease activity and soil sucrase activity during the harvest period of Chinese chives, which was similar to that of Liu et al.^55^. The appropriate application of N, P, and K and the appropriate release of nutrients promoted the growth of crop roots, improved the soil microbial environment, secreted more enzymes and enzyme-promoting substances, and increased soil enzyme activity^56^. In addition, through the analysis of the correlation between soil enzyme activity and soil fertility factors in different harvest periods of Chinese chives, it was found that soil urease, catalase, and sucrase had a great influence on soil nutrients (Fig. 7). The results showed that different soil enzymes had specificity and commonality to soil fertility. The overall activity of soil enzymes can reflect the level of soil fertility, which is shown by the fact that soil enzymes can promote the transformation and absorption of N, P, and K in soil^57^. In the present study, we found that the correlation between soil enzyme activity and soil fertility factors in different harvest periods of Chinese chives was very different, and the correlation between soil enzyme activity and soil nutrients gradually increases during the Chinese chive harvest period (Fig. 7). The relationship between soil enzyme activity and soil nutrients is different in different crops. Soil enzyme is sensitive to changes caused by environmental or management factors, and the level of soil enzyme activity reflects the transformation ability of soil nutrients to a certain extent^58^. The application of SRF can improve the soil available nutrients in the middle and later stages of Chinese chive harvest, ensure the soil metabolic activity, and improve the soil nutrient supply capacity over the entire harvest.

The climatic condition of Wushan was stable, with an annual average temperature of 9.6 ℃ and an annual precipitation of 500 mm. From 2017 to 2018, the average temperature in greenhouse was 12.4 °C, and the average soil temperature in the 0–20 cm plough layer was 12.6 °C. Under stable climatic conditions, soil nutrients in multi-layer covered plastic greenhouses was key factors affecting the quality of wintering Chinese chives. We found that there were significant differences in soil nutrient content and Chinese chive quality among different fertilization treatments. The soil nutrient content of SRF and DSRF treatments was high, and the quality of Chinese chives was higher than that of local conventional fertilization TF treatment. In the present study, the relationship between soil nutrients and the quality of Chinese chives was complicated^59–61^. However, there has been no published research data on the relationship between vegetable quality and soil nutrients. In the present study, the principal component analysis method was used to screen the content of available P and total P that differed greatly among different fertilization treatments, and its effect on the quality of wintering Chinese chives further explored (Fig. 8). The results showed that available P was significant correlation with the soluble protein content of Chinese chives (*p* < 0.01), and was significant correlation with the content of flavonoids, total phenols, and nitrate (*p* < 0.05) (Fig. 9). The content of total P was significantly correlated with the vitamin C, soluble sugar, and soluble protein of Chinese chives (*p* < 0.05), and was significantly correlated with the content of flavonoids and total phenols (*p* < 0.01) (Fig. 9). These results indicated that different soil nutrients have certain effects on the quality variables of Chinese chives, and this effect may be synergistic or antagonistic. It also illustrated the complexity of the relationship between soil nutrients and the quality of Chinese chives. This study used multivariate and quantitative statistical analysis methods to study the soil nutrient content that leads to the highest quality of Chinese chives. We found that, when the available P and total P content in the soil were 125.07 mg kg^−1^ and 1.26 g kg^−1^, respectively, the highest Chinese chive quality can be obtained (1.84 mg kg^−1^ soluble protein, 2.46 OD_325_ g flavonoids, and 0.53 OD_280_ g total phenol) (Fig. 10). During the winter production of Chinese chives in greenhouse in Wushan, the application of P fertilizer can be appropriately increased, which may improve the quality of Chinese chives.

Our results suggested that the application of SRF and decreasing the amount of fertilizer, the frequency of fertilization could be reduced, the effective nutrient content and supply capacity of the soil could be significantly increased, and the soil enzyme activity could be improved. Simultaneously, the content of soluble sugar, soluble protein, and flavonoids in leaves were significantly increased, the nitrate content was significantly reduced. In summary, a decrease of 31% in SRF (DSRF) could promote the growth of Chinese chives and improve soil fertility, thereby enhancing the productivity and quality of Chinese chives.

## Materials and methods

### Experimental sites, greenhouse microclimate, and experiment materials

**Figure 11:**
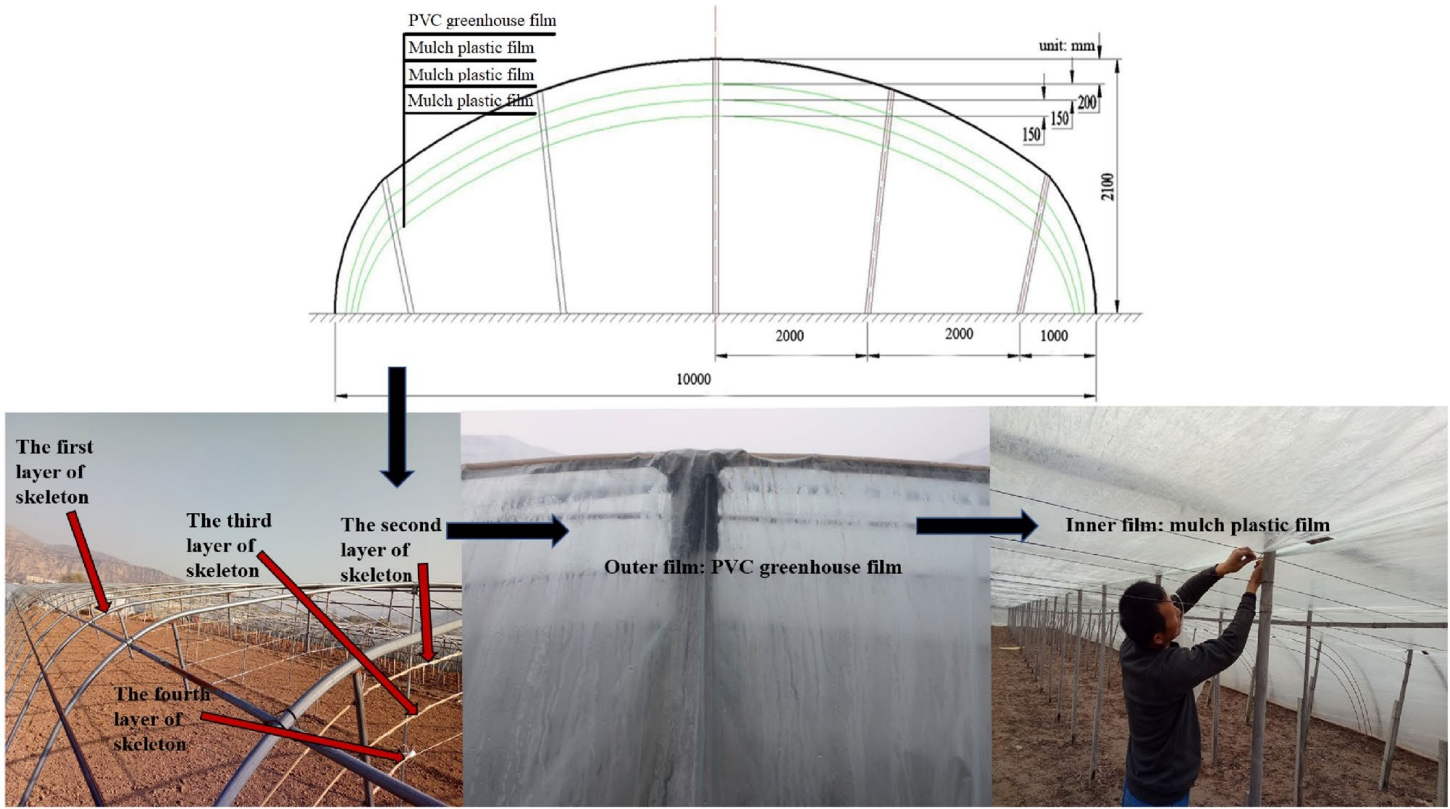
Production mode of the multi-layer (four-layer) covered plastic greenhouse.

**Figure 12:**
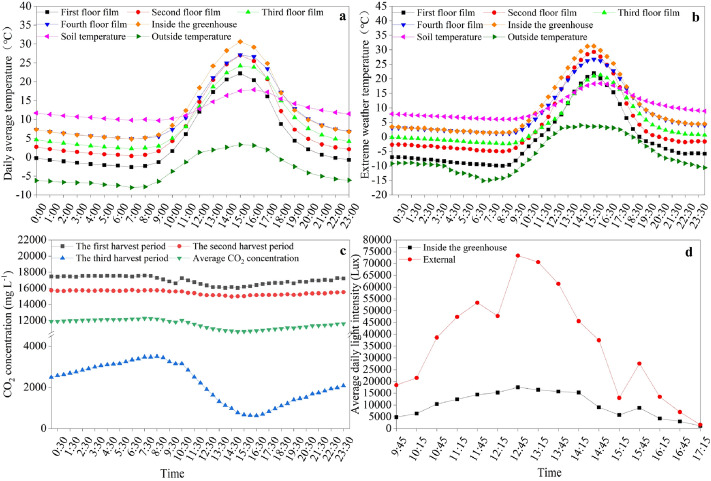
The greenhouse microclimate during the winter production period from December 2017 to February 2018. (**a**) Average temperature; (**b**) extreme weather temperature; (**c**) CO_2_ concentration during winter production period; (**d**) average light intensity.

The experiments were performed during the Chinese chive growing seasons (2017–2018) in Wushan County (N 34^◦^25′–34^◦^57′, E 104^◦^34′–105^◦^08′), in northwest China, with the Chinese chive cultivar ‘Chive God F1′. The production mode of the multi-layer (four-layer) covered plastic greenhouse in Fig. 11. The outer film of the greenhouse is a PVC greenhouse film, and the inner mulch is a three-layer Mulch plastic film. Data on the greenhouse microclimate were gathered using installed environmental monitoring devices^62^, and was presented in Fig. 12. Wushan belongs to a temperate continental semi-humid monsoon climate, with annual average temperature and precipitation of 9.6 °C and 500 mm respectively. The annual evaporation capacity was 1000–2000 mm, and the frost-free period was 120–220 days. The soil type was sandy loam. The soil in the plough layer (0–20 cm) was collected by a soil drill, and its nutrient content was determined^63^. The basic agrochemical properties of the soil were: pH 5.18, EC 2.35 ms cm^−1^, organic matter 15.62 g kg^−1^, available N 54.83 mg kg^−1^, available P 70.20 mg kg^−1^, and available K 121.71 mg kg^−1^. Slow-release fertilizer, a resin-coated compound fertilizer, was used in the experiment. The nutrient release cycle of SRF was 120 days, and the N:P:K in SRF was 26:11:11. Urea (46% N) was used as N fertilizer, calcium superphosphate (12% P) was used as P fertilizer, and potassium sulfate (50% K) was used as K fertilizer.

### Methods and treatments

Chinese chive seedlings were planted on 20 June 2017. The plot size was 10 m × 30 m. The rooting period and production period of Chinese chives were June 20, 2017-November 20, 2017 and November 20, 2017-February 24, 2018, respectively. The greenhouse was buckled with inner film (three-layer Mulch plastic film) and outer film (PVC greenhouse film) on November 20, 2017. The experiment was conducted in a completely randomized design with three replications of four treatments:

(i) No fertilizer (CK);

(ii) Traditional fertilization (TF, NPK dosage 1369.5 kg ha^−1^), applied in accordance with the traditional, customary amount of fertilizer used by local farmers (Rooting period: 298.5 kg N ha^−1^, 394.5 kg P ha^−1^, and 46.5 kg K ha^−1^; Harvest period: 357.0 kg N ha^−1^, 165.0 kg P ha^−1^, and 108.0 kg K ha^−1^);

(iii) Traditional fertilization with slow-release fertilizer (SRF, NPK dosage 1369.5 kg ha^−1^), in which SRF replaced TF, and the nutrient dosage was the same as that of TF;

(iv) Decreased fertilization with slow-release fertilizer (DSRF, NPK dosage 942.0 kg ha^−1^). The total nutrient content in the DSRF was 31% lower than that in the TF (a 33% decrease in N, a 68% decrease in P, and a 110% increase in K). Rooting period: 219.0 kg N ha^−1^, 90.0 kg P ha^−1^, and 162.0 kg K ha^−1^; Harvest period: 219.0 kg N ha^−1^, 90.0 kg P ha^−1^, and 108.0 kg K ha^−1^.

The method for calculating the amount of fertilizer used in DSRF treatment, and the management of water and fertilizer for each treatment were as previously described^62^. The Chinese chives were harvested three times, on January 20, February 8, and February 26, 2018. All other greenhouse management practices were strictly consistent in all treatments.

### Morphological and physiology parameters

During the first harvest of Chinese chives, twenty-seven Chinese chives were randomly picked from each plot within each treatment to determine the plant height, leaf length, stem diameter, chlorophyll content, and the dry matter content (including roots, stems, and leaves). The plant height (the height from the base of the stem to the growth point, cm) and leaf length (the length of the leaf above the pseudo stem, cm) were determined with tape measure; the stem diameter (the diameter from the base of the stem to the middle of the first leaf, mm) was measured with a vernier caliper^64^. The chlorophyll content was determined by the acetone extraction method^65^. Then, the Chinese chives were dried at 105 °C for 30 min, followed by drying at 75 °C until the weight was constant to determine the dry matter content^66^. The plant dry matter increment in different growth periods was calculated to attain the dry matter accumulation rate according to the method described by Zhang et al.^67^. 

### Nutritional quality

During the first harvest, the Chinese chive samples were selected by the sampling method mentioned above to determine the quality of Chinese chives. The 2,6-dichloroindophenol stain method was used to determine the vitamin C content^68^. The soluble sugar content was determined according to the method of Pan et al.^69^. The soluble protein content was measured using the coomassie brilliant blue method^70^. Total phenol and flavonoid content was determined according to Alina et al.^71^ with minor modification. Nitrate content was determined according to Cataldo et al. with a slight modification^72^.

### Soil nutrients and related enzyme activities

The soil was sampled in triplicate to the depth of 0–20 cm plough layer from each plot within each treatment using an auger on January 17, February 6, and February 24, 2018, and the soil was air-dried to determine the soil nutrients and the activities of soil-related enzymes. Soil total N content was measured using the Kjeldahl method, soil total P content was measured using the molybdenum blue colorimetric method, and soil total K content was measured using the flame spectrophotometer method^73,74^. Available N content was measured using potassium permanganate titration^75^. Available P was measured using the ultraviolet absorption method^76^. Available K was measured using the flame photometer method^77^. Soil urease activity was measured using sodium phenol-sodium hypochlorite colorimetry, soil catalase activity was measured using the potassium permanganate titration method, and soil invertase activity was measured using the 3,5-dinitrosalicylic acid colorimetry method^78,79^.

### Statistical analysis

The data were analyzed using IBM SPSS Statistics 21.0 (SPSS Inc., Chicago, IL, USA) and treatment means were separated by Duncan’s multiple range test (*p* < 0.05). Pearson correlation coefficients were calculated for soil nutrients and soil enzymes, and Soil nutrients and quality of Chinese chive data integration using IBM SPSS Statistics 21.0 (SPSS Inc., Chicago, IL, USA). All data were presented as mean ± standard error. Heat map of Pearson's correlation coefficient matrix, principal component analysis (PCA), bar and line diagrams were constructed using OriginPro 2019b 32Bit. The response value of the multiple regression equation was analyzed by design-expert 10.0.7.
